# Variation of antibacterial and antioxidant secondary metabolites and volatiles in leaf and callus extracts of Phulai (*Acacia Modesta* Wall.)

**DOI:** 10.1186/s12870-024-04747-9

**Published:** 2024-02-07

**Authors:** Noura Sh. A. Hagaggi, Usama M. Abdul-Raouf, Tarek A. A. Radwan

**Affiliations:** Botany Department, Faculty of Science, Aswan University, Aswan, 81528 Egypt

**Keywords:** *Acacia modesta*, Antibacterial, Antioxidant, Extract, Secondary metabolites

## Abstract

**Background:**

Acacia species are economically significant as medicinal plants that have been utilized since ancient times. *Acacia modesta* has been reported to possess potent antibacterial and antioxidant properties, but its growth rate is slow. In this study, we hypothesized that inducing callus in vitro from *A. modesta* could enhance the production of antibacterial and antioxidant secondary metabolites, thereby circumventing the issues of slow growth and excessive harvesting of the plant.

**Results:**

The callus was induced from axillary buds on MS medium supplemented with 1 mg/L of 2,4-D and 1 mg/L of BAP. The secondary metabolites, volatile compounds, antibacterial activity, and antioxidant activity of the callus and parent plant leaf extracts were evaluated. The results revealed that the content of phenolics and flavonoids, the number of volatile compounds, and the antibacterial and antioxidant activities of the callus extract were significantly enhanced (*P* ≤ 0.05) compared to the leaf extract. The antibacterial and antioxidant effects were strongly correlated with the total phenolic and flavonoid content in the extracts.

**Conclusions:**

Our findings suggest that in vitro callus culture increases the production of phenolics, flavonoids, and volatile compounds. This subsequently enhances the antibacterial and antioxidant properties of *A. modesta.*

## Background

Global infection control is still at risk due to the spread of antibiotic-resistant pathogens that have developed resistance mechanisms. This issue affects countries at all stages of development, particularly in terms of their healthcare systems [1]. Additionally, free radicals pose a constant threat to human health. These radicals are produced in our bodies during normal metabolic and physiological processes, leading to oxidative stress and the development of chronic diseases such as cancer, aging, diabetes, and inflammatory diseases [2]. As a result, one of the main objectives of global sustainable development is to support the search for affordable, safe, and effective critical medicines that promote good health [3].

Medicinal plants often serve as effective therapies for various diseases because they contain a variety of secondary metabolites that have significant therapeutic potential [4]. Despite advancements in synthetic drugs, the majority of the world’s population still relies on plant-based products [5]. However, the production of bioactive metabolites from plants is limited by geographical and environmental factors [6]. Additionally, plants take a long time to develop and become suitable for producing desired metabolites [7]. To address these challenges, plant tissue culture offers an alternative method for the quick and efficient production of secondary metabolites in a controlled environment without being limited by seasons [8]. Callus culture is particularly effective in obtaining commercially valuable secondary metabolites as callus can be induced from small plant segments, allowing for the direct isolation of secondary metabolites or pharmaceuticals without harming the entire plant [9].

The *Acacia* species have significant commercial value and have been used as traditional medicines since the beginning of human civilization [10]. Many previous research studies have indicated that the Acacia genus contains phytoconstituents with significant biological effects, including antibacterial, antioxidant, anti-inflammatory, and anti-atherosclerotic properties [11, 12]. The inhibitory activities of leaf extracts from *Acacia nilotica*, *A. seyal*, *A. aroma*, *A. albidia*, *A. mellifera,* and *A. tortilis* against pathogenic Gram-positive and Gram-negative bacteria including *Staphylococcus aureus*, *Listeria monocytogenes*, *Escherichia* coli, *Salmonella* spp. and *Pseudomonas aeruginosa* have been reported [13, 14]. Antioxidant phytochemicals such as phenolics and flavonoids have been identified in many Acacia species, including *A. seyal*, *A. podalyriifolia*, *A. rigidula,* and *A. berlandieri* [15, 16].

*Acacia modesta* (wall.), commonly known as Phulai, is a small perennial tree belonging to the Fabaceae family. It is native to Pakistan and also grows in India. This tree typically reaches a height of 3 to 5 m [17]. Previous research has highlighted the remarkable effectiveness of *A. modesta* against pathogenic bacteria such as *Bacillus subtilis*, *Salmonella* spp., *Escherichia coli*, and *Vibrio cholera*. Additionally, it has demonstrated scavenging activities against free radicals [18]. Despite its strong antibacterial and antioxidant properties, *A. modesta* has a slow growth rate [19]. Given that plant tissue culture is a valuable strategy for obtaining active compounds from plants in a sustainable manner, independent of environmental changes [8], we hypothesize that in vitro callus formation from the *A. modesta* parent plant will enhance the production of bioactive secondary metabolites and volatile compounds and subsequently the biological activities, such as antibacterial and antioxidant effects. Therefore, the objective of this study is to induce callus formation from *A. modesta* axillary buds in vitro and evaluate the secondary metabolites, volatile compounds, as well as the antibacterial and antioxidant activities of callus extract and the parent plant leaf extract.

## Materials and methods

### Plant materials

Fresh leaves and axillary buds were taken from *Acacia modesta*, which was cultivated in the Aswan Botanical Garden in Aswan Governorate, Egypt. The specimen was deposited in the herbarium of the garden under the number 29379. The samples were immediately transported to the plant tissue culture lab at Aswan University for further investigation.

### Callus induction

The axillary buds were sterilized by immersing them in 40% sodium hypochlorite for 25 min, and 70% (v/v) ethanol for 5 min. They were then rinsed three times with sterilized distilled water. The MS basal medium of Murashige and Skoog [20] supplemented with 30 g/L sucrose, a combination of 2,4 dichloro-phenoxy acetic acid (2,4-D) (1 mg/L) and benzyl amino purine (BAP) (1 mg/L), and 8 g/L agar (BD Bacto™) was adjusted to a pH of 5.8 and autoclaved at 121 °C for 20 min. The medium was poured into glass jars with plastic caps in aseptic conditions and left at the laminar flow to solidify. The sterilized buds were then placed on the surface of the MS media and kept at a temperature of 27 ± 1 °C under a photoperiodic lighting cycle of 16 h of light and 8 h of darkness. Subculturing was performed every four weeks for a period of up to three months on fresh MS media until enough calluses were obtained.

The fresh weight (g) of the callus was recorded, and then the callus formation rate (%) and callus efficiency (g/callus) were calculated using the formula provided by Erkoyuncu and Yorgancilar [21],

$$\mathrm{Callus}\;\mathrm{formation}\;\mathrm{rate}\;(\%)=\frac{\mathrm{Number}\;\mathrm{of}\;\mathrm{formed}\;\mathrm{callus}}{\mathrm{Total}\;\mathrm{number}\;\mathrm{of}\;\mathrm{cultured}\;\mathrm{explants}}\times100$$

$$\mathrm{Callus}\;\mathrm{efficiency}\;\left(\text{g}/\text{callus}\right)=\frac{\mathrm{Fresh}\;\mathrm{weight}\;\mathrm{of}\;\mathrm{callus}\times\mathrm{Callus}\;\mathrm{formation}\;\mathrm{rate}}{100}$$

### Extraction

Both the leaves of the parent plant and the calluses were air-dried separately. After that, the samples were ground into a powder. The powdered materials (50 g) were then mixed with methanol in a 1:5 (w/v) ratio and stirred for 24 h at room temperature. After centrifuging and filtering the mixtures, the solvent was allowed to evaporate. The obtained extracts were air-dried and weighed, and the percentage yield of each extract was calculated using the formula of El Mannoubi [22]. The extracts were then stored in the refrigerator at 4 °C for further analysis.

### Evaluation of secondary metabolites

#### Total phenolics

The total phenolic content of the extracts was determined using the Folin-Ciocalteu assay [23]. The total phenolic content was calculated as µg of gallic acid equivalents per gram of the extract using the gallic acid standard curve equation: Y = 0.0078X + 0.1861, *R*^2^ = 0.9926, where Y is the absorbance at 700 nm and X is the gallic acid concentration (µg/mL).

#### Total flavonoids

The colorimetric method using aluminum chloride [24] was used to estimate the total flavonoid content of the extracts. The total flavonoid content per gram of the extract was calculated as µg quercetin equivalents using the quercetin standard curve equation: Y = 0.0031X + 0.0159, *R*^2^ = 0.9997, where Y is the absorbance at 510 nm and X is the quercetin concentration (µg/mL).

#### Total saponins

The total content of saponins was evaluated in the extracts by a spectrophotometric assay using vanillin-sulphuric acid reagent [25]. The absorbance was measured at 560 nm using a T60 UV/Vis spectrophotometer. The equation of the standard diosgenin curve: Y = 0.0005X—0.0052, *R*^2^ = 0.9885, was used to calculate the total content of saponins as µg of diosgenin equivalents per gram of the extract, where Y is the absorbance at 560 nm and X is the concentration of diosgenin (µg/mL).

#### Total condensed tannins

The spectrophotometric method described by Sun et al. [26] was used to determine the total tannin content in the extracts. This method involved using a methanolic vanillin reagent. The total tannin content was calculated using the equation of the standard curve of tannic acid: Y = 0.0932X + 0.0672, *R*^2^ = 0.998, where Y is the absorbance at 500 nm and X is the concentration of tannic acid (µg/mL).

#### Determination of volatile profiles

The volatile compounds in the extracts were analyzed using an Agilent Technologies Gas Chromatograph (7890A GC) coupled with a Mass Selective Detector (5977A MSD). The compounds were separated by a TR-5MS GC column (Thermo Scientific™), which has a length, inner diameter, and film thickness of 30 m, 0.25 mm, and 0.25 μm respectively. The carrier gas used was helium at a flow rate of 1.0 mL/min. The initial temperature was set to 60 °C and held for 5 min. Then, the temperature was increased at a rate of 10 °C per minute until it reached 240 °C. It was then maintained at 240 °C isothermally for 20 min. To identify the volatile compounds, their retention indices, and mass spectra were compared to those of the standard mixture of n-alkanes (C_7_ –C_40_) from “Millipore Sigma™ Supelco™” and the NIST standard reference database.

#### In vitro antibacterial activity

The antibacterial effect of the extracts against five pathogenic bacteria was tested following the CLSI standards [27]. The tested bacteria were *Escherichia coli* (ATCC 25922), *Enterobacter cloacae* (ATCC13047), *Klebsiella pneumoniae* (ATCC4352), *Staphylococcus aureus* (ATCC 25923), and *Micrococcus luteus* (ATCC 4698), which were obtained from the reference stock cultures of our Bacteriology Lab at Aswan University. Under aseptic conditions, the tested bacteria were inoculated onto Mueller–Hinton plates, and 6 mm agar wells were made. The extracts were diluted to a concentration of 1 mg/mL in 10% dimethyl sulfoxide (DMSO), and then 50 µL was added to each well. DMSO (10%) and Ampicillin (1 mg/mL) were used as negative and positive controls, respectively. After 24 h of incubation at 37 °C, the diameters (mm) of the inhibition zones were measured.

#### Minimum inhibitory concentration (MIC) of the extracts

The MICs of the extracts were determined following the NCCLS standard [28]. In glass tubes, 5 mL of Mueller–Hinton broth was supplemented with the extracts at final concentrations of 0.25, 0.5, 1, 2, and 4 mg/mL. Each tube was inoculated with 10 µL of a freshly prepared suspension (10^7^ CFU/mL) of the tested bacteria and incubated at 37 °C for 24 h. The MIC was defined as the lowest concentration of the extract at which visible growth was inhibited.

### In vitro antioxidant activity

#### Total antioxidant activity

The phosphomolybdenum spectrophotometric assay [29] was used to evaluate the antioxidant activity of the extracts. In brief, 1 mL of the extract was mixed with 1 mL of the reagent [H_2_SO_4_ (0.6 M), Na_2_HPO_4_ (28 mM), and (NH_4_)_6_Mo_7_O_24_ (4 mM)]. The reaction mixture was incubated for 90 min in a water bath set at 95 °C. After cooling, the color that developed was measured at 695 nm. The total antioxidant activity of the extract was calculated as ascorbic acid equivalents using the equation derived from the ascorbic acid standard curve: Y = 0.0003X + 0.2806, *R*2 = 0.9793, where Y is the absorbance at 695 nm and X is the concentration of ascorbic acid (µg/mL).

#### Radical scavenging activity

The ability of the extracts to scavenge free radicals was estimated using 2,2-diphenyl-1-picrylhydrazyl (DPPH), as described by Najafabad and Jamei [30]. One milliliter of 0.1 mM freshly prepared DPPH methanolic solution was added to 1 mL of the extract and allowed to sit for 30 min at room temperature in the dark. The DPPH solution was used as a control. The absorbance was measured at 517 nm, and the percentage of scavenging was calculated using the following formula:

$$\mathrm{Scavenging}\;\mathrm{activity}\;\left(\%\right)=\frac{\mathrm{Ab}\;\mathrm{control}-\mathrm{Ab}\;\mathrm{extract}}{\mathrm{Ab}\;\mathrm{control}}\times100$$

where Ab is the absorbance at 517 nm.

### Data analysis

The significant differences between the extracts derived from leaf and callus were determined by a student's t-test at a significance level of *P* ≤ 0.05. This analysis was conducted using SPSS software (v.25.0). The values presented are the means of three biological replicates with standard errors. A correlogram was constructed using the R package corrplot (v. 3.4.3) to display Pearson's correlation matrix between the content of secondary metabolites, antibacterial activity, and antioxidant activity in the extracts.

## Results and discussion

**Fig. 1:**
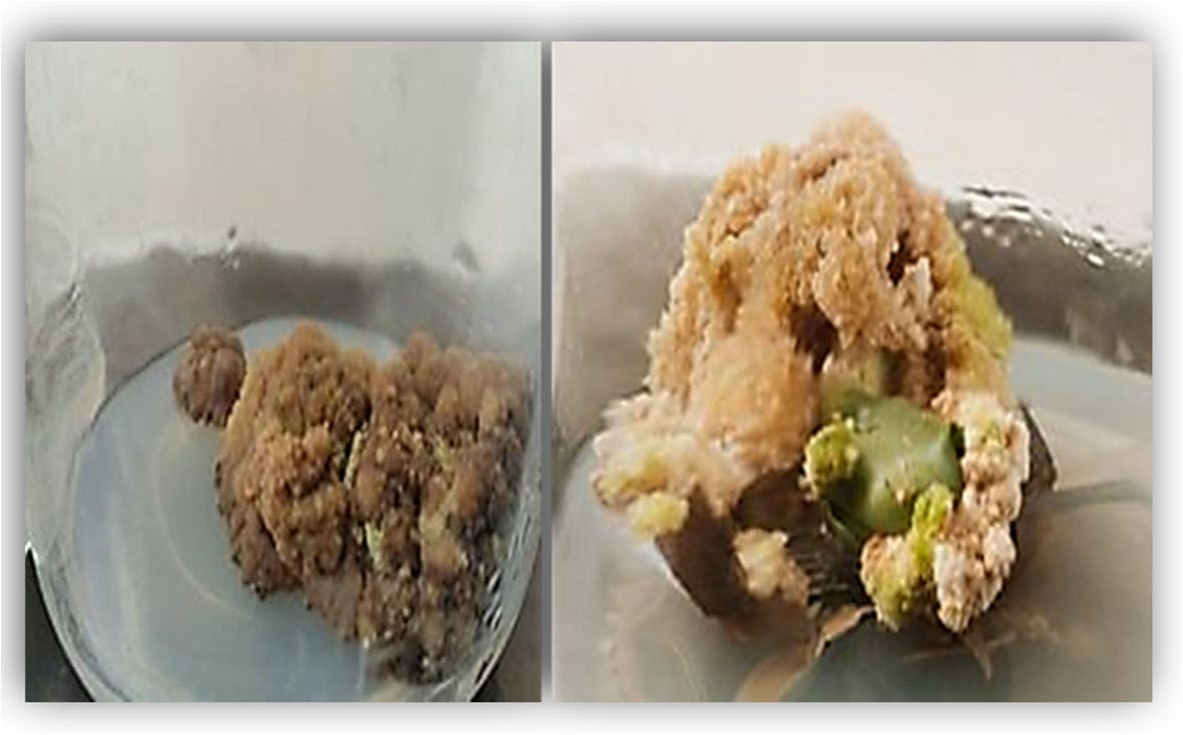
Shows the axillary bud-derived callus of *A. modesta* on MS medium supplemented with 2,4-D (1 mg/L) and BAP (1 mg/L)

In vitro culture is an efficient alternative method for obtaining medicinally significant plant metabolites [31]. This study aimed to investigate the impact of in vitro-cultivated calluses of *A. modesta* on the production of secondary metabolites, volatile compounds, and antibacterial and antioxidant activities in comparison to the leaves of the parent plant. Calluses were successfully induced from the axillary buds of *A. modesta* using MS medium supplemented with a mixture of 2,4-D (1 mg/L) and BAP (1 mg/L) (Fig. 1). To achieve higher production of bioactive secondary metabolites through in vitro plant tissue culture, it is crucial to attain desirable biomass production [32]. In this study, the average values for callus fresh weight (g), callus formation rate (%), and callus efficiency (g/callus) were 4.7, 75, and 3.53, respectively.

### Contents of secondary metabolites in the extracts

**Fig. 2:**
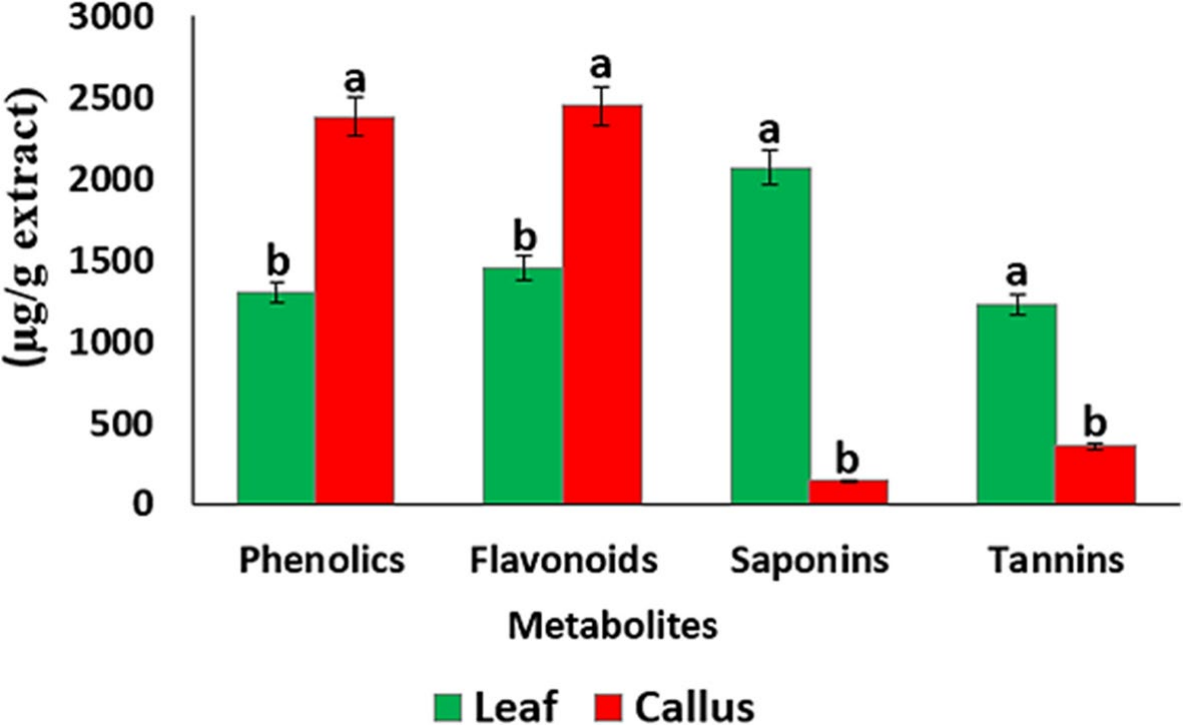
Evaluation of secondary metabolites in leaf and callus extracts of *A. modesta*

The leaf and callus materials were extracted with methanol, and extraction yields of 28.8% and 33.2% were obtained, respectively. The data analysis revealed significant differences in the secondary metabolite contents in the leaf and callus extracts (*p* < 0.00001). The leaf extract contained phenolic and flavonoid concentrations of 1302 ± 2.8 and 1450 ± 3.5 µg/g extract, respectively, while the callus extract had 2384 ± 2.8 and 2449 ± 2.1 µg/g extract, respectively (Fig. 2). This difference may be attributed to the fact that the callus was developed in a medium supplemented with plant growth regulators, which have been shown to affect the synthesis and accumulation of phenolic and flavonoid compounds [33]. On the other hand, the leaf extract had saponins and tannins of 2070 ± 1.4 and 1225 ± 2.8 µg/g extract, respectively, compared to the callus extract, which contained 144 ± 1.4 and 358 ± 1.4 µg/g extract (Fig. 2). Saponins and tannins play a significant role in protecting plants against microbial infections, herbivores, and insects [34, 35]. Since the callus developed in a controlled environment, it may not need to synthesize large amounts of saponins and tannins. In contrast, saponins and tannins in the leaf may be accumulated as a defense response against biotic and abiotic stresses [36].

### GC–MS volatile profiles of the extracts

**Fig. 3:**
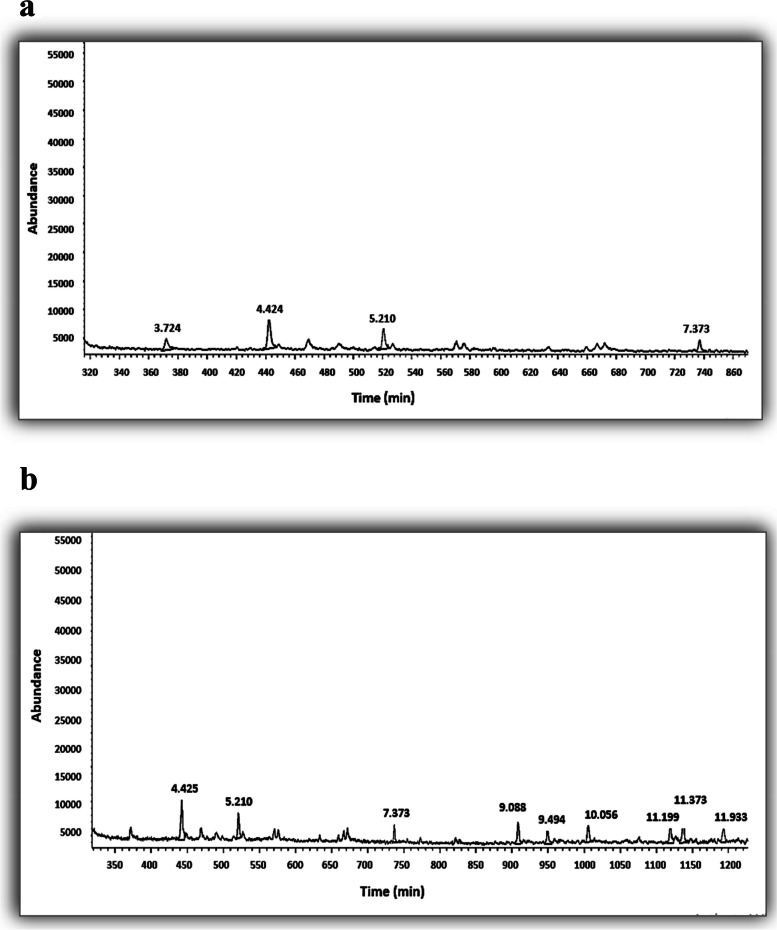
GC–MS chromatograms of the extracts: leaf extract (**a**) and callus extract (**b**)

The GC–MS analysis detected four compounds in the leaf extract, (1S)-2,6,6-Trimethylbicyclo [3.1.1] hept-2-ene, Heptane, 2,2,4,6,6-pentamethyl-, 1-Octadecane sulphonyl chloride and Oxalic acid, hexyl tetradecyl ester with area percentages of 17.168%, 43.114%, 26.003% and 13.715%, respectively (Table 1, Fig. 3a). On the other hand, nine compounds were identified and quantified in the callus extract: Heptane, 2,2,4,6,6-pentamethyl (20.855%), 2,6-Dimethyldecane (11.069%), Undecane, 3,7-dimethyl- (6.186%), tert-Butyl dimethyl silyl nitrile (11.708%), Undecane, 3,7-dimethyl- (8.289%), Di-n-decyl sulfone (9.503%), Oxalic acid, hexadecyl hexyl ester (10.734%), Oxalic acid, 6-ethyloct-3-yl heptyl ester (13.571%) and Hexane, 2,2,3,3- (8.084%) (Table 2, Fig. 3b). Numerous studies have previously indicated that in vitro culture stimulates the production of volatile organic compounds in plants [37, 38].

**Table 1:** GC–MS volatile profile of *A. modesta* leaf extract

No	Retention time	Area%	Compound	Molecular formula	Molecular weight	CAS NO
1	3.724	17.168	(1S)-2,6,6-Trimethylbicyclo [3.1.1] hept-2-ene	C_10_H_16_	136	7785–26-4
2	4.424	43.114	Heptane, 2,2,4,6,6-pentamethyl-	C_12_H_26_	170	13475–82-6
3	5.210	26.003	1-Octadecane sulphonyl chloride	C_18_H_37_ClO_2_S	352	1000342–70-4
4	7.373	13.715	Oxalic acid, hexyl tetradecyl ester	C_22_H_42_O_4_	370	1000309–24-8

**Table 2:** GC–MS volatile profile of *A. modesta* callus extract

No	Retention time	Area%	Compound	Molecular formula	Molecular weight	CAS NO
1	4.426	20.855	Heptane, 2,2,4,6,6-pentamethyl	C_12_H_26_	170	13475–82-6
2	5.210	11.069	2,6-Dimethyldecane	C_12_H_26_	170	13150–81-7
3	7.373	6.186	Undecane, 3,7-dimethyl-	C_13_H_28_	184	17301–29-0
4	9.090	11.708	tert-Butyl dimethyl silyl nitrile	C_7_H_15_NSi	141	56522–24-8
5	9.496	8.289	Undecane, 3,7-dimethyl-	C_13_H_28_	184	17301–29-0
6	10.057	9.503	Di-n-decyl sulfone	C_20_H_42_O_2_S	346	111530–37-1
7	11.201	10.734	Oxalic acid, hexadecyl hexyl ester	C_24_H_46_O_4_	398	1000309–25-0
8	11.373	13.571	Oxalic acid, 6-ethyloct-3-yl heptyl ester	C_19_H_36_O_4_	328	1000309–34-5
9	11.933	8.084	Hexane, 2,2,3,3-	C_10_H_22_	142	13475–81-5

### In vitro antibacterial activity and MIC values

**Fig. 4:**
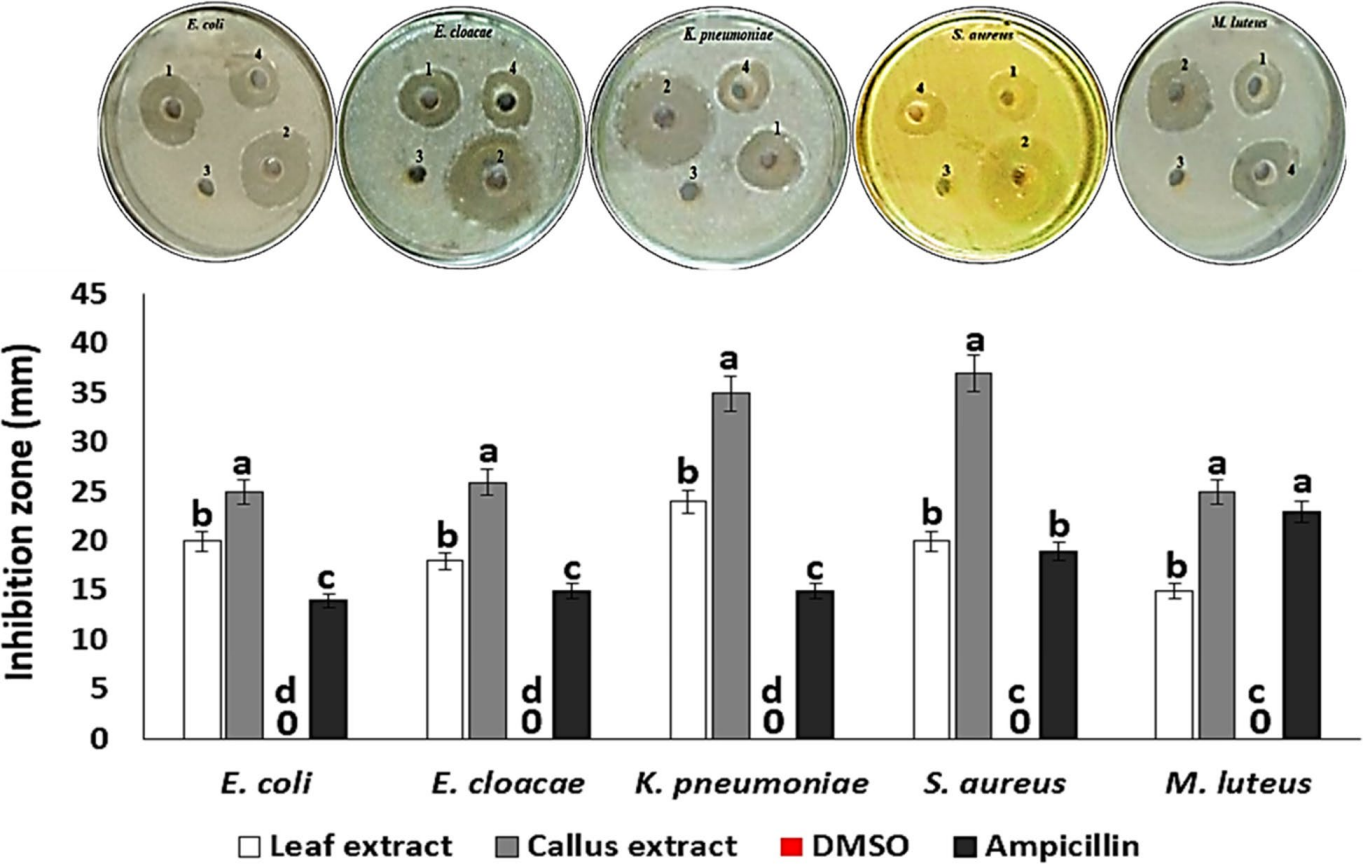
In vitro antibacterial activities of *A. modesta* leaf and callus extracts. The numbers on the Petri dishes represent the following: 1 for leaf extract, 2 for callus extract, 3 for 10% DMSO (negative control), and 4 for 1 mg/mL ampicillin (positive control). The small letters on the column chart indicate a significant difference at *P* ≤ 0.05

**Fig. 5:**
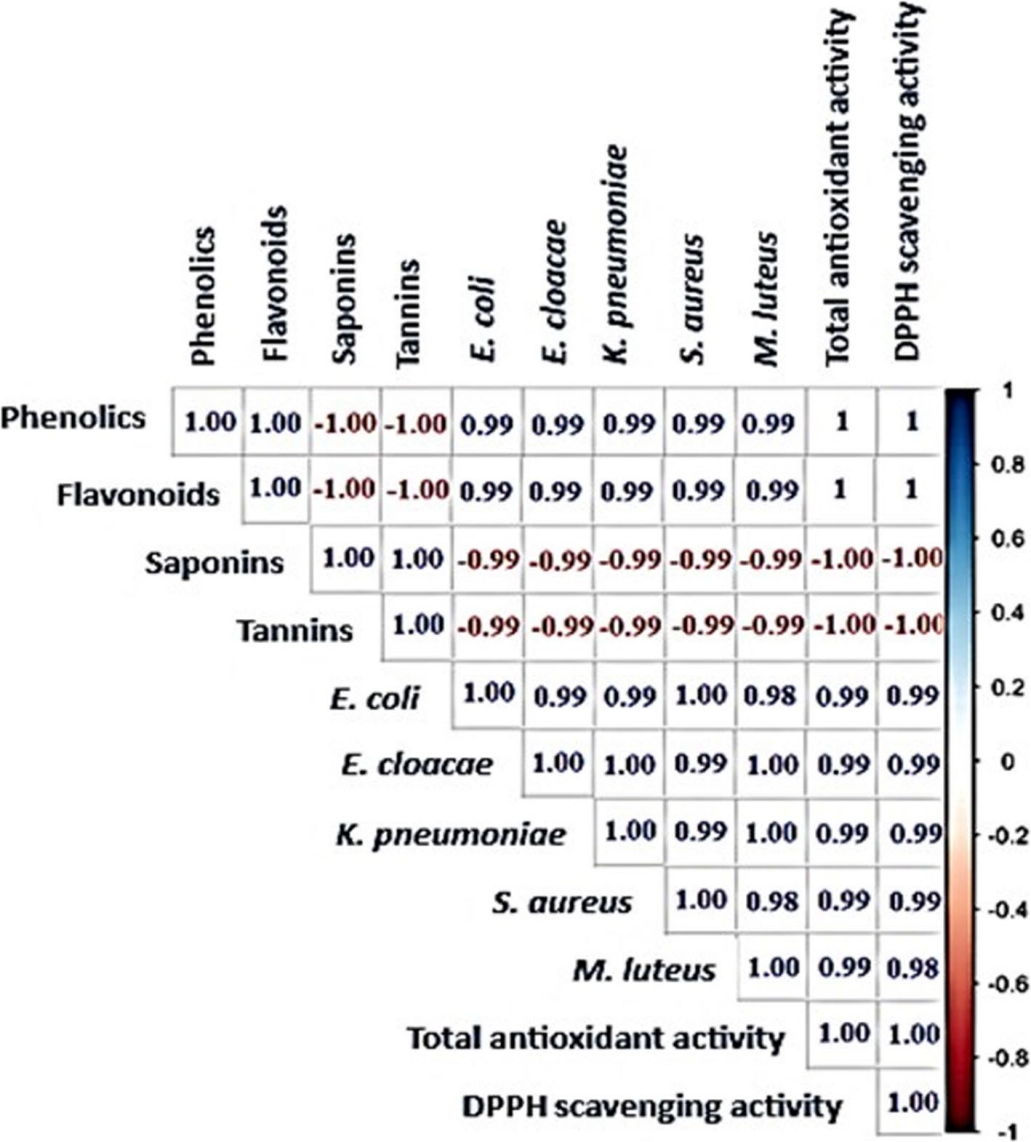
A correlogram displaying Pearson's correlation coefficients (*r*) among secondary metabolite content, antibacterial activity, and antioxidant activity of the extracts. Strong positive correlations were found between the antibacterial activity, antioxidant activity, and the levels of phenolics and flavonoids in the extracts, with correlation coefficient (*r*) values of 0.99 and 1.00, respectively. Strong negative correlations were observed between the antibacterial activity, antioxidant activity, and the levels of saponins and tannins in the extracts, with correlation coefficient (*r*) values of -0.99 and -1.00, respectively

Five pathogenic bacterial species, including *E. coli*, *E. cloacae*, *K. pneumoniae*, *S. aureus*, and *M. luteus,* were selected for this study because they are known to be involved in many human diseases [39, 40]. Although both the leaf and callus extract showed inhibitory activity against all the tested pathogenic bacteria, there were significant differences in the antibacterial activity (*P* = 0.0009) and MIC values (*p* = 0.002) between the leaf and callus extracts for each tested bacterial strain. The callus extract was found to be more potent than the leaf extract (Table 3, Fig. 4). This study is the first attempt to induce calluses from *A. modesta* in vitro, so there are no previous reports on the antibacterial activity of callus extracts from *A. modesta*. However, our findings are consistent with previous studies, that have reported a broader spectrum of antibacterial activity of *A. modesta* leaf extract against various pathogenic bacteria including *Bacillus subtalis*, *Entereococcus faecalis*, *Staphylococcus aureus*, *Mycobacterium tuberculosis*, *Pseudomonas aeruginosa*, *Klebsiella pneumoniae*, *Micrococcus luteus*, *Escherichia coli*, and *Salmonella typhi* [41]. The relationship between the content of secondary metabolites and the antibacterial activity of the extracts was analyzed using R package corrplot based on Pearson's correlation coefficient (Fig. 5). A positive correlation (*r* = 0.99) was observed between the total contents of phenolics and flavonoids in the extracts and their antibacterial activity (Fig. 5). Previous studies have reported that phenolics and flavonoids exhibit antibacterial activity by inhibiting nucleic acid biosynthesis and metabolic activities of bacterial cells [42, 43]. Additionally, the majority of volatile compounds detected in the callus extract are hydrocarbons (C_n_H_2n+2_) (Table 2), which have been shown to possess antibacterial properties [44].

**Table 3:** In vitro antibacterial activity and minimum inhibitory concentration (MIC) values of *A. modesta* leaf and callus extracts

Pathogenic bacteria	Leaf extract		Callus extract		DMSO (negative control)	Ampicillin (positive control)
	**Inhibition zone (mm)**	**MIC (mg/mL)**	**Inhibition zone (mm)**	**MIC (mg/mL)**	**Inhibition zone (mm)**	
*Escherichia coli*	20 ± 0.0^b^	1 ± 0.0^a^	25 ± 0.7^a^	0.5 ± 0.28^b^	0.0 ± 0^d^	14 ± 0.1^c^
*Enterobacter cloacae*	18 ± 1.4^b^	1 ± 0.002^a^	26 ± 0.7^a^	0.5 ± 0.0^b^	0.0 ± 0^d^	15 ± 0.0^c^
*Klebsiella pneumoniae*	24 ± 1.4^b^	1 ± 0.01^a^	35 ± 0.7^a^	0.25 ± 0.14^b^	0.0 ± 0^d^	15 ± 0.0^c^
*Staphylococcus aureus*	20 ± 0.0^b^	0.5 ± 0.0^a^	37 ± 0.7^a^	0.25 ± 0.14^b^	0.0 ± 0^c^	19 ± 1.4^b^
*Micrococcus luteus*	15 ± 1.4^b^	1 ± 0.14^a^	25 ± 0.0^a^	0.5 ± 0.0^b^	0.0 ± 0^c^	23 ± 0.7^a^

The values are the means of three replicates ± standard errors

The superscript small letters indicate a significant difference at *P* ≤ 0.05

^a,b^Indicate the presence of significant differences between leaf and callus extracts

### In vitro antioxidant activity

**Fig. 6:**
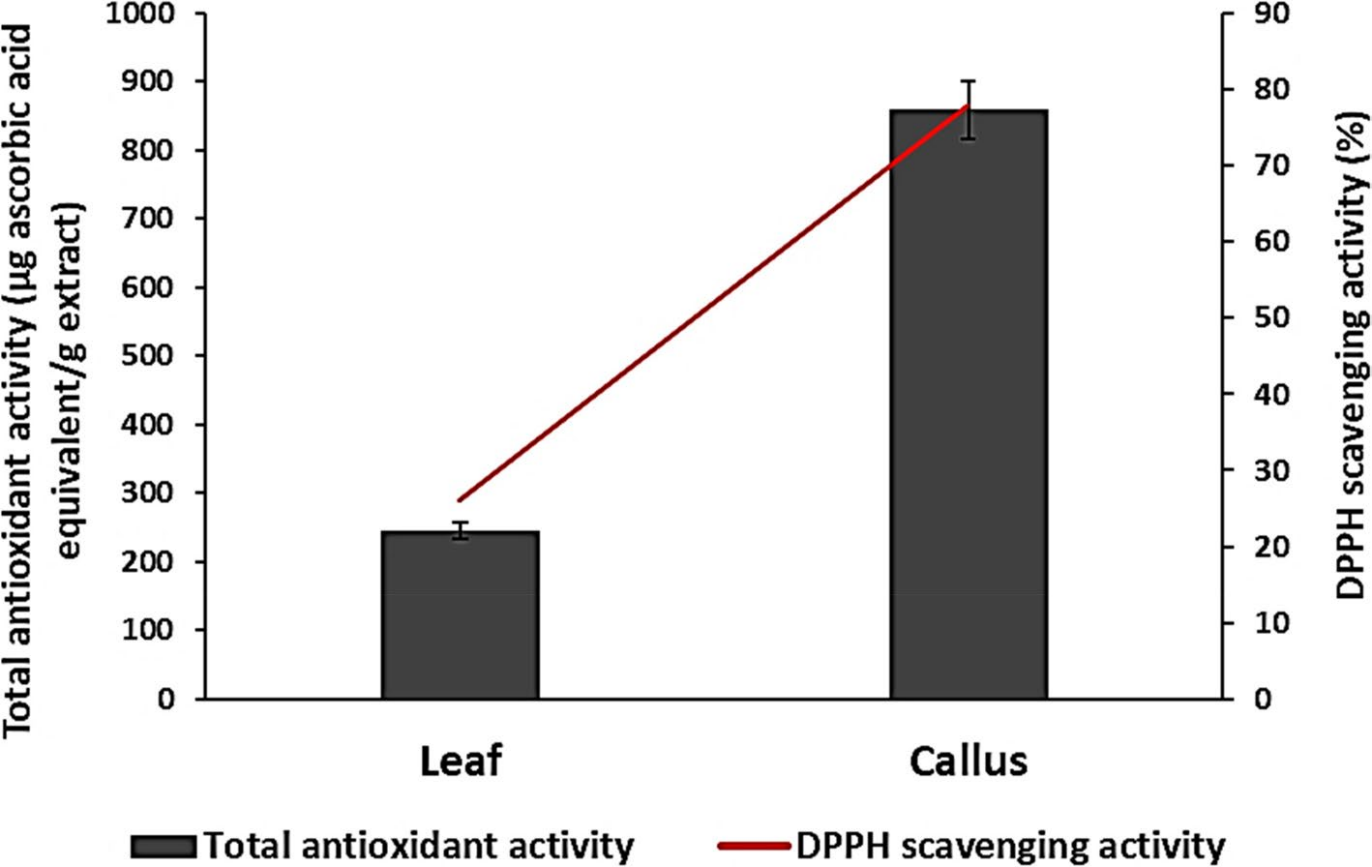
In vitro antioxidant activity of leaf and callus extracts from *A. modesta*

Various reactions occur in the human body, leading to the accumulation of free radicals that cause cell damage and diseases [45]. Antioxidants can scavenge free radicals, reducing or eliminating their harmful effects [46]. Synthetic antioxidants are harmful to human beings [47]. Plant-derived antioxidants, on the other hand, have been proven to be useful in protecting human health [48]. Therefore, this study investigated the total antioxidant activity of *A. modesta* leaf and callus extracts as well as their ability to scavenge DPPH radicals in vitro. The total antioxidant activity of the callus extract (859 ± 1.4 µg ascorbic acid equivalents/g extract) was significantly (*p* < 0.00001) higher than that of the leaf extract (246 ± 2.1 µg ascorbic acid equivalents/g extract). Additionally, the callus extract showed a significant increase (*p* < 0.00001) in DPPH scavenging activity by 52% compared to the leaf extract (Fig. 6). Pearson’s correlation coefficient analysis revealed a strong positive correlation (*r* = 1) between the antioxidant activities of the extracts and their phenolic and flavonoids contents (Fig. 5). This finding is consistent with other studies that have found a positive correlation between antioxidant activity, phenolics, and flavonoids [49, 50]. It has been previously reported that many species of Acacia such as *A. modesta*, *A. seyal*, *A. sieberiana*, *A. nilotica*, *A. ataxacantha*, *A. auriculiformis*, and *A. crassicarpa*, are known for their strong antioxidant activities [51, 52].

**Fig. 7:**
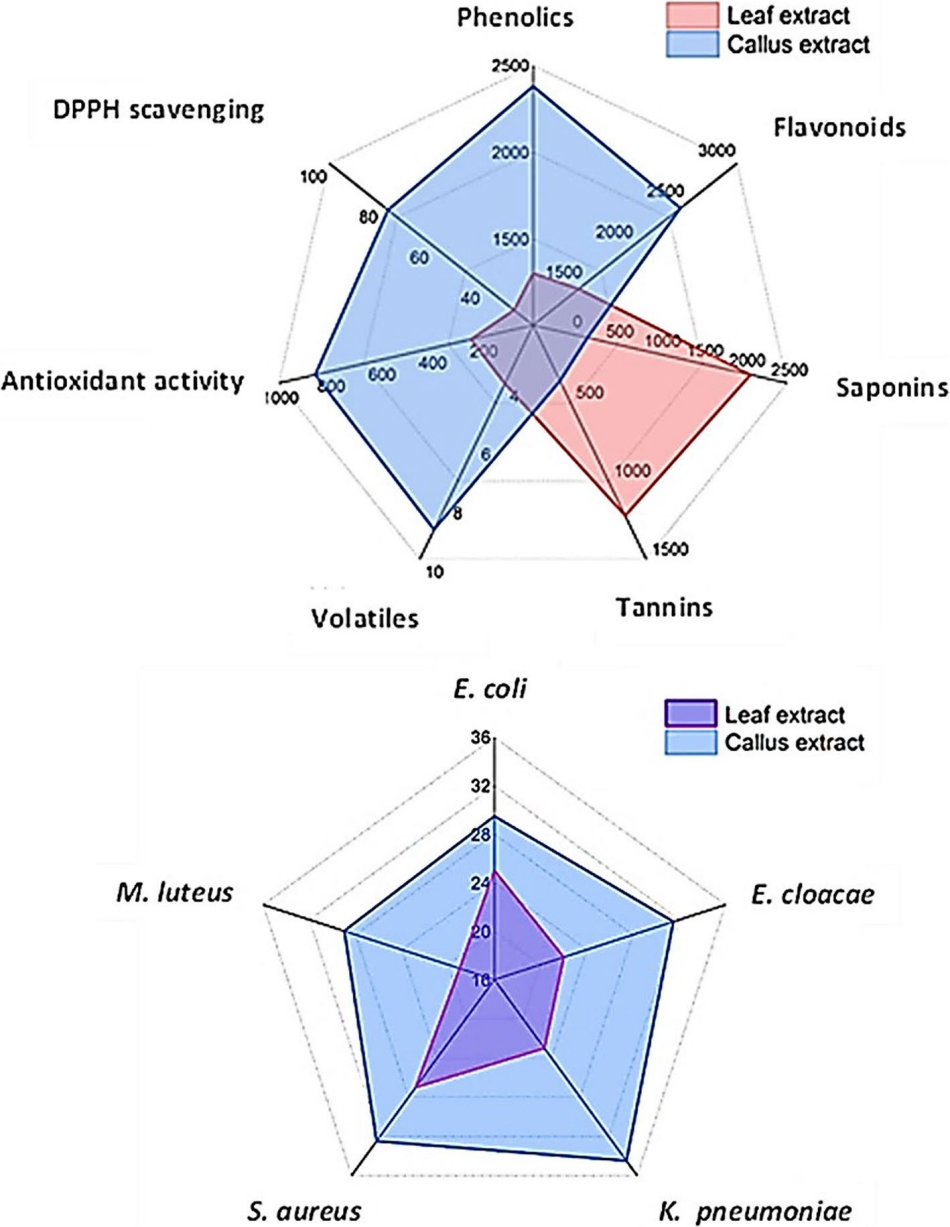
Radar plot illustrating the differences in secondary metabolite content, volatiles, as well as antibacterial and antioxidant activities between leaf and callus extracts of *A. modesta*

Finally, the results of this study are summarized in Fig. 7. The figure displayed significant variations in secondary metabolite content, the number of detected volatile compounds, and antibacterial and antioxidant activity between the leaf and callus extracts. It was revealed that the callus extract is the most potent. These findings align with previous studies, which have indicated that callus culture has the potential to produce more biologically active secondary metabolites compared to wild plants. This suggests that callus culture can be utilized as a source of bioactive substances in the fields of medicine and pharmaceuticals [53–55].

## Conclusions

This study aims to find a way to utilize the antibacterial and antioxidant properties of *Acacia modesta* while avoiding the issues of slow growth and over-exploitation, which could lead to the species becoming endangered. Callus was successfully induced from axillary buds using MS medium supplemented with 2,4-D and BAP. The results of the study confirmed that the callus extract had higher levels of phenolics, flavonoids, volatile compounds, and antibacterial and antioxidant properties compared to the parent leaf extract. Therefore, callus culture could be used for large-scale production of valuable antibacterial and antioxidant secondary metabolites in *A. modesta*.

## Data Availability

The datasets generated during the current study are available from the corresponding author upon reasonable request.
